# Microsurgical Management of Carotid Body Tumors: An Educational Neurosurgical Perspective with Video Demonstrations

**DOI:** 10.3390/jcm15072508

**Published:** 2026-03-25

**Authors:** Abdullah Keles, Ufuk Erginoglu, Yerkebulan Serikanov, Yannick Canton Kessely, Sima Sayyahmelli, Oyku Ozturk, Nafiye Sanlier, Behman Demir, Maryam Sabah Al-Jebur, Umid Sulaimanov, Mustafa Kemal Baskaya

**Affiliations:** Department of Neurological Surgery, University of Wisconsin School of Medicine and Public Health, Madison, WI 53792, USAerginoglu@wisc.edu (U.E.); serikkanov@wisc.edu (Y.S.); sayyahmelli@neurosurgery.wisc.edu (S.S.); nafiyesanlier@gmail.com (N.S.); aljebur@wisc.edu (M.S.A.-J.); sulaimanov@wisc.edu (U.S.)

**Keywords:** balloon test occlusion, carotid body tumor, head and neck tumors, microsurgical resection, paraganglioma, preoperative embolization

## Abstract

**Background/Objectives:** Carotid body paragangliomas, commonly referred to as Carotid Body Tumors (CBTs), are rare, highly vascular paragangliomas arising at the carotid bifurcation and pose significant surgical challenges due to their proximity to critical neurovascular structures. Optimal management remains debated, particularly for large or complex lesions. This study aims to present a structured neurosurgical operative workflow as an educational and practical resource to help young surgeons understand operative decision-making and technical execution from a neurosurgical perspective. **Methods:** We retrospectively reviewed patients diagnosed with CBTs and identified three cases that underwent microsurgical resection by a single neurosurgeon. Clinical presentation, radiographic findings, operative strategies, intraoperative microsurgical techniques, and postoperative outcomes were analyzed. Surgical procedures for all three cases are further illustrated with technical video demonstrations highlighting meticulous microsurgical techniques performed by a single neurosurgeon. **Results:** All three patients presented with either incidental or slowly progressive neck masses, with imaging demonstrating classic splaying of the internal and external carotid arteries. One patient exhibited elevated catecholamine metabolites, while another had a familial history of paragangliomas. Preoperative embolization was successfully performed in all three cases. Complete tumor resection was achieved in each patient. One patient developed post-embolization embolic ischemic changes with transient neurological deficits that were resolved within several hours. No permanent cranial nerve deficits, vascular injuries, or tumor recurrences were observed. Pathology confirmed paraganglioma in all cases. **Conclusions:** Surgical resection remains an effective treatment for CBTs, which are commonly managed by vascular or head and neck surgeons. This case series illustrates the technical feasibility of CBT resection using a comprehensive neurosurgical strategy that integrates endovascular preparation, cerebral perfusion assessment, and meticulous microsurgical technique. Rather than proposing novel surgical innovation, this report aims to provide a structured operative framework and detailed video-based illustration of complex carotid bifurcation management from a neurosurgical perspective.

## 1. Introduction

The carotid body is a small chemoreceptor organ measuring approximately 5 × 3 × 2 mm, located in the adventitia of the posteromedial surface of the carotid bifurcation. It is derived from third branchial arch mesodermal elements and neural crest ectoderm, with mesodermal elements giving rise to chemoreceptor cells and neural crest cells differentiating into paraganglionic cells. The carotid body regulates pulmonary ventilation to maintain arterial pO_2_, pCO_2_, and pH homeostasis through afferent input via the glossopharyngeal nerve to the medullary reticular formation. It is primarily responsive to hypoxia, hypercapnia, and acidosis, with stimulation resulting in increased respiratory rate and tidal volume [1,2,3]. Although it receives its blood supply predominantly from vessels of the external carotid artery (ECA), mainly the ascending pharyngeal branch, the internal carotid artery (ICA), vertebral artery, and thyrocervical trunk may also provide blood supply [1,2,3].

Carotid body paragangliomas, commonly referred to as Carotid Body Tumors (CBTs), are rare, highly vascular, and slow-growing benign tumors originating from the paraganglia of the carotid bodies. They are the most common paragangliomas of the head and neck. They occur mainly between fourth and fifth decades and more frequent in females than males (1.9:1). Their incidence is one in 30,000 [3,4,5]. They are mainly non-secreting tumors, but rarely they secrete catecholamines, the metabolites of which can be identified in urine as metanephrines and vanillylmandelic acid [2,6]. Rarely (%5), they metastasize, most commonly to the regional lymph nodes; metastasis to distant organs has also been reported [2,7].

Clinically, CBTs most commonly present as firm, painless, palpable neck masses that are mobile laterally but not vertically (Fontaine sign) [2,8]. Larger tumors may cause neck discomfort, hoarseness, dysphagia, stridor, tongue weakness, and dizziness due to compression of surrounding neurovascular structures, most frequently involving the vagus and hypoglossal nerves. Additionally, CBTs may rarely cause Horner Syndrome and present with cerebral ischemia [2,3].

Radiologic confirmation is essential for diagnosis and surgical planning and typically includes ultrasound, computed tomography (CT), magnetic resonance imaging (MRI), and angiography [9]. CT and MRI can define the extent of the lesions and help in planning surgery [4]. The “lyre sign” is characteristic and shows splaying of the ICA and the ECA. In case of suspected CBT, bilateral cerebral angiography should be performed. It helps to evaluate arterial supply, concurrent atherosclerosis, contralateral carotid arteries, and collateral flow.

Surgical removal is the first choice of treatment. In patients with advanced age, multiple comorbidities, or bilateral CBTs with poor outcomes after surgical removal on one side might be considered for conservative treatment or radiosurgery [2,5]. Historically, neurosurgeons were frequently involved in surgical procedures involving the carotid bifurcation, particularly in conjunction with carotid endarterectomy and other carotid artery operations. However, with the widespread adoption of endovascular techniques for carotid stenosis, neurosurgeons have become less frequently involved in open surgery of the carotid complex. As a result, CBTs are now more commonly managed by vascular or head and neck surgeons. In most reported series, tumor excision is performed using conventional open surgical techniques, typically with direct visualization or loupe magnification. Although operative microscopes have been described in selected reports to enhance visualization during tumor dissection, their routine use in CBT surgery has not yet been widely adopted [2,10,11,12,13].

Herein, we present a structured neurosurgical operative framework for the microsurgical management of three patients with CBTs. Rather than proposing novel surgical techniques, this report aims to illustrate the integration of preoperative embolization, balloon test occlusion (BTO) with hypotensive challenge, multimodal intraoperative neuromonitoring, and meticulous subadventitial microsurgical dissection into a cohesive operative strategy. Through detailed technical descriptions and high-quality video demonstrations, we highlight operative decision-making, neurovascular preservation principles, and multidisciplinary coordination in the management of complex carotid bifurcation pathology.

## 2. Results

### 2.1. Case 1

A 42-year-old man presented to our neurosurgery outpatient clinic with a self-identified right-sided neck mass that he had noticed approximately ten years earlier. He had initially been evaluated at an outside otolaryngology clinic, where a neck CT confirmed the presence of a lesion. The patient reported that the mass may have slowly increased in size over time. He denied pain, dysphagia, weight loss, fevers, or night sweats. He was a nonsmoker.

**Figure 1:**
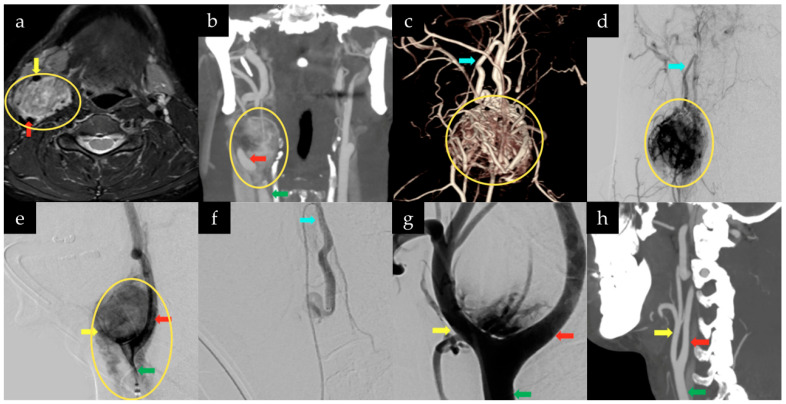
Multimodal imaging of a 42-year-old man with a Shamblin Type 3 right CBT treated with preoperative embolization and microsurgical resection. (**a**) Axial contrast-enhanced MRI demonstrates a hypervascular mass (yellow circle) at the right carotid bifurcation with displacement of the internal carotid artery (ICA, red arrow) and external carotid artery (ECA, yellow arrow). (**b**) Coronal CT angiography shows separation of the ICA (red arrow) and ECA (yellow arrow) by the tumor (yellow circle), with the common carotid artery (CCA, green arrow) identified proximally, consistent with circumferential carotid encasement. (**c**–**e**) Three-dimensional CT angiographic reconstruction and digital subtraction angiography demonstrate extensive tumor (yellow circle) related neovascularity arising predominantly from external carotid artery branches, including the ascending pharyngeal artery (blue arrow), with circumferential involvement of the ICA, ECA, and proximal CCA. (**f**,**g**) Selective and common carotid artery angiography following embolization demonstrate marked reduction in tumor vascularity with preserved patency of the ICA, ECA, and CCA. (**h**) Three-month postoperative CT angiography confirms complete tumor resection with preserved carotid artery anatomy and no evidence of residual or recurrent disease.

The neck CT demonstrated a hypervascular soft-tissue mass splaying the ICA and ECA at the carotid bifurcation, consistent with a CBT (Figure 1). Physical examination revealed a palpable right-sided neck mass and was otherwise unremarkable. The patient denied any neurological symptoms. He had no significant past medical or surgical history.

The patient reported a family history notable for bilateral CBTs in his father, for which no intervention had been performed.

The risks of surgical resection were reviewed in detail, and preoperative embolization of the tumor on the day prior to surgery was recommended to reduce intraoperative blood loss. 24-h urine studies for catecholamines and metanephrines were within normal limits.

Further imaging demonstrated a heterogeneously enhancing soft-tissue mass splaying the right carotid bifurcation, classified as a Shamblin type 3 lesion, most consistent with a CBT. Several small arterial branches arising from the ECA were noted to supply the mass. Additionally, a small 6 × 6 mm nodule within the left carotid space, interposed between the ICA and ECA, was identified and considered indeterminate but suspicious for a second paraganglioma. There was no evidence of flow-limiting stenosis, aneurysm, or vascular malformation.

Surgical resection with preoperative embolization was planned.

Under general anesthesia, diagnostic angiography with BTO and hypotensive challenge was performed and successfully passed. This was followed by endovascular embolization of the tumor.

The next day, under general anesthesia and continuous neuromonitoring, the patient was positioned supine with a shoulder roll, the neck extended, and the head turned to the left. A linear incision was made along the right neck anterior to the sternocleidomastoid muscle. At this stage, the operative microscope was introduced to provide enhanced magnification, illumination, and stereoscopic visualization for the microsurgical dissection. Under high magnification, dissection proceeded medially to the sternocleidomastoid muscle to identify the CCA. At the carotid bifurcation, a large, complex tumor was encountered. The surrounding soft tissue was significantly more fibrotic and vascular than anticipated, prolonging the dissection required to expose the CCA and tumor.

The carotid sheath was ultimately opened. The common facial vein was ligated, and the external jugular vein was mobilized out of the operative field. Two large nerves traversing the superior aspect of the exposure were identified and meticulously protected throughout the procedure. Dissection of the tumor began at its inferior aspect along the CCA. Inspection revealed that the tumor encased both the ICA and ECA. Intraoperative Doppler ultrasound confirmed preserved flow within both vessels.

Gentle circumferential dissection was performed using a combination of bipolar electrocautery, sharp dissection, and controlled traction to peel the tumor from the CCA, the ICA followed by the ECA. Multiple feeding vessels originating directly from the carotid bifurcation were identified and divided.

The most challenging portion of the dissection involved a tumor adherent to the carotid bifurcation and posterior aspect of the CCA. Due to limited visualization, the ICA and ECA were rotated approximately 180 degrees to allow direct visualization and safe removal of the tumor from this region. The tumor was ultimately freed completely from the carotid bifurcation. Meticulous hemostasis was achieved, and the wound was closed in layers. Continuous intraoperative electroencephalography (EEG), motor evoked potentials (MEPs), and somatosensory evoked potentials (SSEPs) monitoring revealed no changes throughout the procedure (Video S1). Estimated intraoperative blood loss was approximately 30 mL. The duration of the procedure was approximately 3 h from skin incision to final closure. Postoperative examination revealed no cranial nerve deficits. The patient was discharged home on postoperative day 2. Final pathology confirmed paraganglioma with benign lymph node tissue.

At the three-month postoperative visit, the patient denied any new neurological symptoms. Computed tomography angiography (CTA) of the head and neck demonstrated no residual or recurrent tumor and no evidence of flow-limiting stenosis, aneurysm, or vascular malformation. The left carotid space nodule remained stable in size and was felt to likely represent a second paraganglioma. Long-term radiographic surveillance with magnetic resonance angiography is planned. At six months following surgery, the patient continues to remain neurologically intact without clinical evidence of recurrence.

### 2.2. Case 2

A 51-year-old woman was evaluated for stroke-like symptoms, consisting of right arm weakness, right leg weakness, and facial asymmetry. Imaging revealed no evidence of acute infarction; however, an incidental left carotid body lesion was identified. Laboratory evaluation was unremarkable except for elevated urinary dopamine output. Aspirin therapy was initiated following the stroke-like episode.

At presentation to our institution, the patient denied any neurological or systemic symptoms. Her past medical history was significant for depression, anxiety, irritable bowel syndrome, hypertension, type 2 diabetes mellitus, obesity, migraines, and hearing loss. Her surgical history included hysterectomy, bladder sling surgery, gastric bypass, abdominoplasty, appendectomy, and cholecystectomy. She had a strong family history of hypertension. Physical examination at admission was unremarkable.

**Figure 2:**
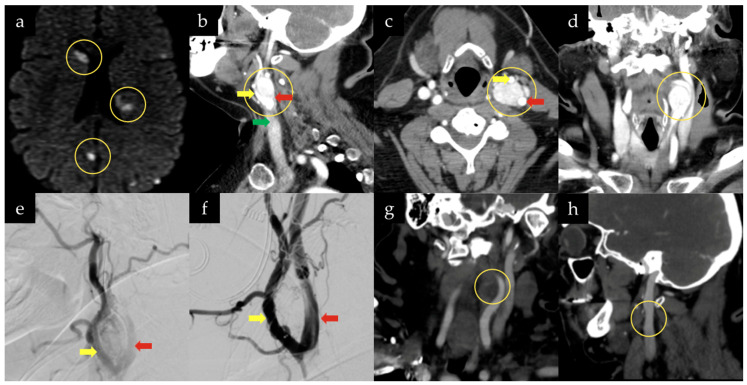
Multimodal imaging of a 51-year-old woman with a left carotid body tumor treated with preoperative embolization and surgical resection. (**a**) Diffusion-weighted MRI demonstrates multifocal areas of restricted diffusion (yellow circles) within the supratentorial cerebral hemispheres, more prominent on the left, including involvement of the caudate head and putamen, consistent with acute multifocal ischemic infarctions without evidence of intracranial hemorrhage. Sagittal (**b**), axial (**c**), and coronal (**d**) contrast-enhanced CT angiography of the neck demonstrate a hypervascular mass centered at the carotid bifurcation (yellow circle) with a circumferential relationship to the cervical carotid vasculature and delineate the craniocaudal extent of the lesion. (**e**) Preoperative digital subtraction angiography demonstrates marked tumor vascularity supplied predominantly by branches of the external carotid artery, with substantial reduction in tumor blush following selective embolization (**f**) and preserved patency of the internal and external carotid arteries. Postoperative coronal (**g**) and sagittal (**h**) CT angiography confirm complete tumor resection without residual or recurrent lesion and preservation of carotid artery anatomy (yellow circles). (ICA, red arrow; ECA, yellow arrow; CCA, green arrow).

CTA of the neck demonstrated a hypervascular mass at the left carotid bifurcation, encasing the ICA and ECA. The lesion extended from the level of the carotid bifurcation at C3–4 to the superior aspect of the second cervical vertebral body. There was no evidence of flow-limiting stenosis in either vessel (Figure 2).

Given the size and vascularity of the lesion, surgical resection following preoperative embolization was selected as the preferred treatment strategy. A BTO was planned preoperatively in anticipation of potential carotid artery clamping. Following embolization, definitive surgical resection was scheduled for the following day.

The highly vascular nature of the tumor and the associated risk of intraoperative hemorrhage were discussed in detail with the patient and her husband, who agreed to proceed. Due to the risk of vascular injury, graft material was made available intraoperatively in case vascular reconstruction or patch angioplasty became necessary.

Under general anesthesia with continuous EEG, MEPs, and SSEPs monitoring, the patient successfully passed the BTO with hypotensive challenge. The tumor was embolized via branches of the ascending pharyngeal artery using particulate embolization, without procedural complications. No changes in EEG, MEPs, or SSEPs were observed during the procedure.

Postoperatively, the patient was transferred to the neurosurgical intensive care unit (NICU). Overnight, she developed hoarseness and right-hand weakness (4-/5 strength). Diffusion-weighted MRI demonstrated multiple embolic infarcts involving the caudate nucleus and posterior limb of the internal capsule.

On the following day, the patient was taken to the operating room for open microsurgical resection of the lesion.

Under general anesthesia with continuous EEG, MEPs, and SSEP monitoring, a curvilinear incision was made along the anterior border of the sternocleidomastoid muscle. At this stage, the operative microscope was introduced to provide enhanced magnification and illumination for the meticulous microsurgical dissection. Dissection proceeded to identify the internal jugular vein, common facial vein, and carotid artery. A large vascular mass was visualized at the carotid bifurcation, with prominent feeders arising from the base of the bifurcation.

Superficial dissection was initiated along the most prominent portion of the mass. Embolization material was noted at the base of the ICA. Circumferential dissection was performed using a combination of bipolar coagulation and sharp dissection. Despite prior embolization, the tumor remained moderately vascular intraoperatively, requiring careful hemostatic control during circumferential dissection. The mass was ultimately delivered en bloc. Upper and lower cervical lymph nodes were also excised. Meticulous hemostasis was achieved, and the wound was closed in layers. No pathological changes were observed on intraoperative neuromonitoring throughout the surgery (Video S2). Estimated intraoperative blood loss was approximately 200 mL. Operative time totaled approximately 2.5 h from the initial incision to wound closure. At postoperative follow-up, there were no cranial nerve deficits.

The patient was discharged to a rehabilitation unit on postoperative day 7. Final pathology revealed a paraganglioma with benign lymph nodes. At the 3-month postoperative follow-up, the patient’s right-hand weakness had completely resolved, and follow-up MRI demonstrated no evidence of tumor recurrence. At six years and eight months of follow-up, there remained no radiographic evidence of recurrent paraganglioma.

### 2.3. Case 3

**Figure 3:**
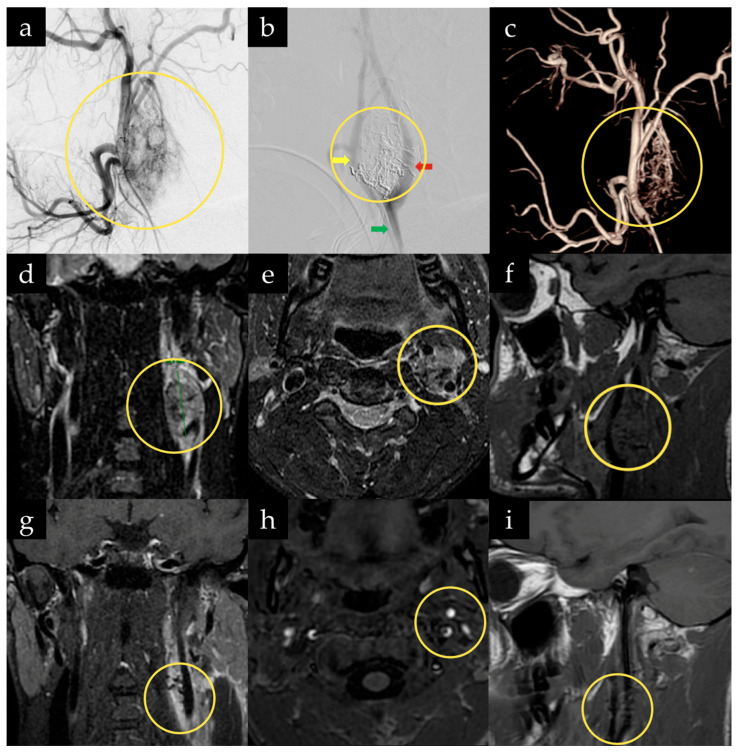
Multimodal imaging of a 24-year-old man with a left carotid body tumor treated with preoperative embolization and surgical resection. (**a**) Preoperative digital subtraction angiography demonstrates a highly vascular tumor (yellow circle) centered at the carotid bifurcation with prominent arterial feeders, with (**b**) marked reduction in tumor (yellow circle) vascularity following embolization. (**c**) Three-dimensional angiographic reconstruction illustrates complex tumor-associated neovascularity (yellow circle) arising from branches of the external carotid artery. Coronal (**d**), axial (**e**), and sagittal (**f**) contrast-enhanced T1-weighted MRI demonstrate the craniocaudal extent of the hypervascular mass (yellow circles) and its relationship to adjacent cervical structures. Postoperative coronal (**g**), axial (**h**), and sagittal (**i**) contrast-enhanced T1-weighted MRI confirm gross total resection with no residual enhancement and preservation of regional anatomy (yellow circles). (ICA, red arrow; ECA, yellow arrow; CCA, green arrow).

A 24-year-old man undergoing evaluation for pneumonia was found to have an incidental left-sided carotid body lesion. His past medical history was significant for Devic’s disease (neuromyelitis optica). Physical examination was unremarkable. Laboratory urinary testing revealed no catecholamine metabolites. MRI demonstrated splaying of the ICA and ECA with enhancing tissue interposed between the two vessels, consistent with a CBT (Figure 3).

A diagnostic cerebral angiography was performed, during which the patient successfully passed a BTO with hypotensive challenge without neurological deficits or EEG changes.

At a follow-up visit, a treatment plan consisting of preoperative embolization followed by open surgical resection the following day was discussed and agreed upon.

The patient subsequently underwent successful embolization of the left-sided carotid body lesion using Onyx. He tolerated the procedure well and was admitted to the NICU for observation. On the following day, he underwent open resection of the lesion with a combined endovascular and open microsurgical team.

Under general anesthesia and continuous neuromonitoring with EEG, MEPs, and SSEPs, the anterior border of the sternocleidomastoid muscle was identified, and a skin incision was made anterior to it. At this stage, the operative microscope was brought into the field, and the dissection proceeded under high magnification and improved illumination. Under the operative microscope, dissection was carried down to expose the CCA, and further distal dissection allowed identification of the tumor. The ICA and ECA, as well as the origin of the superior thyroid artery, were subsequently identified.

The superficial portion of the tumor was carefully dissected away from the CCA and ECA. Several feeding vessels were identified, coagulated, and divided under meticulous hemostasis. The superior thyroid artery was sacrificed to allow lateral rotation of the carotid bifurcation, facilitating further dissection and removal of the posterior aspect of the tumor. The tumor was ultimately resected en bloc. Hemostasis was achieved, and the wound was closed in a standard layered fashion. Intraoperative neuromonitoring revealed no pathological changes throughout the procedure (Video S3). Estimated intraoperative blood loss was approximately 20 mL. The surgery was completed in approximately 3 h, measured from skin incision to closure. No cranial nerve deficits were observed postoperatively.

The patient was discharged home on postoperative day 3. Final pathology demonstrated a paraganglioma with benign lymph nodes. At three years and four months of follow-up, there was no clinical or radiographic evidence of tumor recurrence.

## 3. Discussion

CBTs are rare, highly vascular paragangliomas arising at the carotid bifurcation and continue to present significant surgical challenges despite advances in imaging, endovascular techniques, and intraoperative monitoring. The present cases highlight the variability in clinical presentation, tumor biology, vascular involvement, and surgical complexity of CBTs, underscoring the importance of preoperative planning and meticulous microsurgical technique.

CBTs are slow-growing tumors, and unresected lesions may become increasingly difficult to remove as they enlarge and further involve the carotid complex and lower cranial nerves, leading to higher operative morbidity [14,15,16]. These observations support consideration of surgical intervention before extensive neurovascular encasement develops.

CBTs are classically categorized as sporadic, hereditary, or hyperplastic. Sporadic tumors account for approximately 85% of cases, while hereditary forms represent about 10% and are inherited in an autosomal dominant pattern [3,4,17]. Hyperplastic CBTs are associated with chronic hypoxia, particularly in patients living at high altitude or with chronic pulmonary disease [3,18]. In the present series, one patient demonstrated bilateral carotid space lesions and a positive family history, raising concern for a hereditary paraganglioma. In such cases, the risk of multicentric disease supports the need for long-term radiographic surveillance and consideration of genetic counseling.

Although the differential diagnosis of lateral neck masses includes cervical lymphadenopathy, branchial cleft cysts, lipomas, carotid artery aneurysms, metastatic disease, and nerve sheath tumors, characteristic imaging findings—particularly splaying of the carotid bifurcation—allow for reliable preoperative diagnosis in most cases [2,17]. Any suspicious lesion should undergo radiological evaluation.

CBTs receive their blood supply primarily from branches of the ECA, most commonly the ascending pharyngeal artery, as well as from arterioles within the adventitia of the carotid arteries [19]. Additional contributions from the ICA, vertebral artery, and thyrocervical trunk have also been described [1,2,3].

Preoperative imaging findings such as circumferential carotid encasement on MRI or angiographic evidence of vessel contour irregularity or stenosis may suggest arterial wall invasion, although definitive assessment often requires intraoperative visualization [11].

Surgical resection remains the treatment of choice for most CBTs. However, conservative management or radiotherapy may be appropriate in selected patients, particularly those with advanced age, significant comorbidities, bilateral tumors, or poor tolerance of surgical risk. Small and less vascular lesions can be removed without injury of the cranial nerves and carotid artery. Larger tumors tend to encase surrounding neurovascular structures especially cranial nerves and carotid artery which makes the surgery more difficult. Their hypervascularity makes the surgery challenging, and vascular and neurological sequelae are most common complications of surgical treatment [8,20].

Over the past decades, several surgical strategies have been described for the management of CBTs. Early reports emphasized the technical difficulty and substantial morbidity associated with surgical resection, particularly due to the intimate relationship between the tumor, carotid arteries, and lower cranial nerves. Historical series reported cranial nerve injury rates as high as 32–44% and significant cerebrovascular complications when meticulous vascular control was not achieved [2]. Advances in vascular surgery, improved imaging modalities, and refinements in operative technique have significantly reduced these risks in contemporary series.

Traditional management strategies described in the vascular and head and neck surgery literature typically emphasize early surgical resection with careful subadventitial dissection along the carotid artery wall, identification and preservation of cranial nerves, and proximal and distal vascular control. Surgical planning is commonly guided by the Shamblin classification, which predicts operative difficulty based on the degree of carotid vessel involvement [21]. Modern multidisciplinary management often involves collaboration between vascular surgeons, otolaryngologists, and interventional radiologists to optimize both surgical exposure and vascular control [22].

A major area of ongoing debate in the literature is the role of preoperative embolization. Several authors have reported that embolization may facilitate tumor resection by reducing vascularity and intraoperative blood loss [23]. However, other studies and systematic reviews have failed to demonstrate consistent reductions in operative time, blood loss, or cranial nerve injury rates when embolization is routinely performed [20]. Earlier reports also suggested that embolization may not significantly improve outcomes in medium-sized tumors and may prolong hospitalization due to the additional procedure [24]. Because of these conflicting findings, the use of preoperative embolization varies widely among institutions and is often selectively applied depending on tumor size, vascular supply, and surgeon preference.

More recently, large systematic reviews have further clarified the risks associated with CBT surgery. In a meta-analysis including over 4000 tumors, Robertson et al. reported perioperative stroke rates of approximately 3–4% and cranial nerve injury rates approaching 25%, with increasing risk in higher Shamblin grade tumors [3]. These findings highlight the importance of meticulous operative planning and the continued need for strategies that minimize neurovascular morbidity.

Historically, neurosurgeons were frequently involved in surgical procedures at the carotid bifurcation, particularly in conjunction with carotid endarterectomy and other open carotid artery operations. With the widespread adoption of endovascular stenting techniques for carotid artery disease, neurosurgeons have become less commonly involved in open surgery of the carotid complex. Consequently, CBTs are now more often managed by vascular or head and neck surgeons. In this context, the present series provides a contemporary neurosurgical perspective on the microsurgical management of CBTs, emphasizing principles of cerebrovascular exposure, meticulous microsurgical dissection, and neurovascular preservation.

Compared with previously described management strategies, our operative workflow incorporates several principles derived from contemporary cerebrovascular neurosurgery. First, management was conducted within a multidisciplinary framework involving close collaboration between neurosurgery and neuroradiology teams experienced in endovascular procedures, allowing coordinated preoperative planning and intraoperative decision-making. Second, preoperative tumor embolization was routinely performed to reduce tumor vascularity and facilitate safer microsurgical dissection. Third, when carotid manipulation or temporary occlusion was anticipated, systematic evaluation of cerebral collateral circulation was performed using BTO with hypotensive challenge, providing critical information regarding the patient’s tolerance of potential carotid flow alterations. Fourth, continuous multimodal intraoperative neuromonitoring including EEG, MEPs, and SSEPs was used during carotid manipulation to detect early signs of cerebral ischemia. Finally, tumor removal was performed using meticulous subadventitial microsurgical dissection principles derived from cerebrovascular surgery, emphasizing preservation of the carotid artery complex and adjacent cranial nerves. Collectively, these elements represent a structured neurovascular workflow that integrates endovascular planning, physiologic monitoring, and microsurgical technique to enhance operative safety in the management of carotid body tumors.

Another technical aspect of our operative workflow is the use of operative microscopy during tumor dissection. In many reported series, tumor resections are performed using standard open techniques with direct visualization or loupe magnification. The operative microscope provides high-magnification stereoscopic visualization and improved illumination of the surgical field, which facilitates precise identification of the subadventitial plane and preservation of the carotid arteries and adjacent cranial nerves. Although microscopy has not been universally adopted in CBT surgery, these visualization advantages may support safer microsurgical dissection in tumors involving the carotid bifurcation [2,10,11,12,13].

The present cases illustrate how these principles can be applied in practice during the management of tumors with variable anatomy, vascularity, and operative complexity. In our series, all tumors were successfully resected using microsurgical techniques, combined with preoperative endovascular embolization, while preserving the integrity of the carotid artery complex and surrounding neural structures. Early proximal and distal vascular control, identification and preservation of cranial nerves, and systematic circumferential dissection along the carotid adventitia were key technical principles. Continuous intraoperative neuromonitoring with EEG, MEPs, and SSEPs provided additional safety during carotid manipulation and temporary flow alterations. No intraoperative monitoring changes were observed. Estimated blood loss ranged from 20 to 200 mL, and operative duration was approximately 2.5–3 h. No permanent cranial nerve deficits or vascular injuries were identified postoperatively.

Operative duration reported in the literature varies considerably depending on tumor characteristics and the use of preoperative embolization. Ward et al. [25] reported a mean operative time of 1.75 ± 1.08 h in embolized patients compared with 4.2 ± 1.5 h in non-embolized patients. LaMuraglia et al. [26] described operative times of 4.1 h in embolized cases and 4.5 h in non-embolized cases. Similarly, Li et al. [8] reported mean operative durations of 2.8 ± 1.25 h in embolized patients versus 3.7 ± 1.9 h in non-embolized patients, while Zhang et al. [27] demonstrated median operative times of 3 h in embolized cohorts compared with 3.6 h in non-embolized groups. The operative duration observed in our series is therefore comparable to previously reported embolized cohorts. However, given the small sample size and descriptive design, direct comparison should be interpreted cautiously and not as evidence of comparative superiority.

The impact of preoperative embolization on intraoperative blood loss remains controversial. Multiple studies have shown that preoperative embolization can shorten operative duration, reduce intraoperative blood loss, and decrease complication rates in selected cases [5,9,17,28]. A systematic review and meta-analysis reported no significant difference in mean estimated blood loss between embolized and non-embolized patients [3]. In contrast, Ward et al. reported decreased estimated blood loss, operative time, and cranial nerve injuries in patients undergoing preoperative embolization compared with historical controls, while LaMuraglia et al. observed reduced blood loss in embolized tumors of similar size [25,26]. Wang et al. demonstrated a significant reduction in blood loss among patients with medium-sized tumors measuring 3 to 5 cm, although this difference was not significant when all tumor sizes were analyzed collectively [21]. In our series, estimated blood loss ranged from 20 to 200 mL. While this appears lower than mean values reported in larger cohorts, direct comparison is not appropriate given the small sample size and heterogeneity in tumor characteristics and reporting standards. In our experiences, preoperative embolization facilitated tumor devascularization and improved surgical exposure, which was associated with limited intraoperative blood loss in this small series. Nevertheless, our experience also illustrates the potential risks associated with embolization. One patient developed transient embolic infarcts following embolization despite an uneventful procedure and normal intraoperative neuromonitoring. Embolic complications may occur due to particle migration, reflux into the internal carotid artery, or unrecognized collateral circulation, even in technically successful procedures. This complication highlights the need for thorough counseling regarding the risks of embolization, particularly in tumors with extensive arterial supply or complex angioarchitecture.

Preservation of flow throughout the procedure is of utmost importance to avoid potentially devastating postoperative ischemic complications. In certain cases, temporary occlusion might be necessary, which should not be performed without preoperative evaluation with BTO and hypotensive challenge and without continuous neuromonitoring. If prolonged temporary occlusion is anticipated, then shunt usage should be considered. However, shunts should not be used routinely, as they carry their own risks and should be reserved for cases with neuromonitoring changes. Similarly, if sacrifice of the ICA, ECA, or CCA is a must, then alternative shunting options should be considered and if not possible then leaving residual tumor pieces could be considered if patient fails BTO and hypotensive challenge.

BTO was popularized by Serbinenko in the 1970s [29]. A hypotensive challenge as an adjunct can help with predicting negative BTO but risk of delayed ischemia never falls to zero [30,31]. BTO with hypotensive challenge was performed in all patients and provided valuable information regarding cerebral collateral reserve. Although none of the patients ultimately required carotid sacrifice or reconstruction, having this information preoperatively allowed for contingency planning and improved intraoperative confidence.

The Shamblin classification has traditionally been used to stratify carotid body tumors based on their relationship to the carotid artery and to predict surgical morbidity. In this system, Type 1 tumors are minimally adherent and easily resectable, Type 2 tumors partially encase the carotid vessels and are more challenging to dissect, and Type 3 tumors surround the entire carotid bifurcation and are associated with increased operative risk [1]. However, this classification has important limitations. It does not reliably assess true vessel wall involvement, and tumor size does not consistently correlate with surgical complexity. Therefore, further studies were also attempted to modify Shamblin classification [32].

In our experience, operative difficulty was more strongly influenced by factors such as tumor vascularity, fibrosis, and adherence to the neurovascular structures than by size alone. This was particularly evident in our Shamblin type 3 case, in which extensive fibrosis and posterior vessel wall adherence necessitated circumferential dissection and controlled rotation of the carotid bifurcation to achieve safe tumor removal.

Histopathological examination confirmed benign paraganglioma in all cases, with no evidence of regional lymph node metastasis. No tumor recurrence was observed during follow-up. Consistent with previously described features, tumors were grossly rubbery, red-brown, and well circumscribed. Microscopically, they were composed of nests of epithelioid cells with granular eosinophilic cytoplasm and neural elements within the tumor capsule [2]. These pathological features correlate with the firm yet friable intraoperative consistency and marked vascularity encountered during microsurgical dissection.

Although our series demonstrated favorable operative metrics and neurological outcomes, the small sample size and descriptive design preclude meaningful statistical comparison with larger published cohorts. These findings should therefore be interpreted as illustrative of technical feasibility rather than comparative superiority.

Complications may occur during preoperative embolization, intraoperatively, or postoperatively. Potential complications include ischemic stroke, cranial nerve injury involving cranial nerves IX–XII, recurrent laryngeal nerve palsy, sympathetic chain injury, injury to the mandibular branch of the facial nerve, and carotid artery injury [19]. In a meta-analysis by Robertson et al., cranial nerve XII was the most frequently injured nerve, followed by cranial nerve X, sympathetic nerves, cranial nerve VII, cranial nerve IX, the recurrent laryngeal nerve, the superior laryngeal nerve, and cranial nerve XI. The authors reported a 30-day mortality rate of 2.2%, a stroke rate of 3.5%, a temporary cranial nerve injury rate of 20.4%, a persistent cranial nerve injury rate of 11.2%, and a hematoma rate of 5.2% [3].

## 4. Limitations

This study is limited by its retrospective design, small sample size, and lack of formal comparative outcome analysis. Although operative blood loss, duration, and cranial nerve outcomes are reported, the descriptive nature of this series precludes meaningful statistical comparison with larger published cohorts. Therefore, the findings should be interpreted as illustrative of operative feasibility and workflow rather than comparative efficacy. Nevertheless, the detailed technical descriptions and high-quality video documentation provide insight into surgical decision-making in complex carotid bifurcation pathology.

## 5. Conclusions

Surgical management of CBTs can be performed safely with a low rate of morbidity when appropriate patient selection and meticulous technique are applied. From a neurosurgical perspective, a combined approach involving an experienced, multidisciplinary team is critical to achieving optimal outcomes. In this small case series, preoperative embolization, when used selectively, was associated with limited intraoperative blood loss and facilitated surgical exposure; however, these findings should be interpreted as illustrative of technical feasibility rather than evidence of comparative outcome advantage. Furthermore, meticulous subadventitial microsurgical dissection is essential for minimizing both intraoperative and postoperative vascular complications and cranial nerve injuries.

## Data Availability

The data presented in this study are available on request from the corresponding author due to privacy, legal, or ethical reasons.

## Supplementary Materials

The following supporting information can be downloaded at: https://www.mdpi.com/article/10.3390/jcm15072508/s1, Video S1: Case 1; Video S2: Case 2; Video S3: Case 3.

## Abbreviations

The following abbreviations are used in this manuscript:

CBT	Carotid Body Tumor
ECA	External carotid artery
ICA	Internal carotid artery
CT	Computed tomography
MRI	Magnetic resonance imaging
CTA	Computed tomography angiography
BTO	Balloon test occlusion
EEG	Electroencephalography
MEPs	Motor evoked potentials
SSEP	Somatosensory evoked potential
NICU	Neurosurgical intensive care unit
CCA	Common carotid artery
